# A multicenter phase II study of induction chemotherapy with FOLFOX-4 and cetuximab followed by radiation and cetuximab in locally advanced oesophageal cancer

**DOI:** 10.1038/sj.bjc.6606093

**Published:** 2011-01-18

**Authors:** F De Vita, M Orditura, E Martinelli, L Vecchione, R Innocenti, V C Sileni, C Pinto, M Di Maio, A Farella, T Troiani, F Morgillo, V Napolitano, E Ancona, N Di Martino, A Ruol, G Galizia, A Del Genio, F Ciardiello

**Affiliations:** 1 Division of Medical Oncology, Department of Clinical and Experimental Medicine and Surgery ‘F Magrassi e A Lanzara’, Second University of Naples, Via Pansini 5, Naples 80131, Italy; 2 Division of Radiotherapy, CRO, Via Franco Gallini 2, Aviano (PN) 33081, Italy; 3 Division of Medical Oncology, Azienda Ospedaliera di Padova, Via Nicolò Giustiniani 1, Padova 35128, Italy; 4 Division of Oncology, Azienda Ospedaliera di Bologna, Via Albertoni 15, Bologna 40138, Italy; 5 National Cancer Institute, Clinical Trials Unit, Via Mariano Semmola, 80131 Naples, Italy; 6 Division of Radiotherapy, Federico II University of Naples, Via Pansini 5, Naples 80131, Italy; 7 Division of Surgical Oncology, Department of Clinical and Experimental Medicine and Surgery ‘F Magrassi e A Lanzara’, Second University of Naples, Naples, Italy; 8 Division of Surgery, University of Padova, Via 8 Febbraio 2, Padova 35122, Italy

**Keywords:** oesophageal cancer, preoperative chemoradiotherapy, cetuximab, FOLFOX-4

## Abstract

**Background:**

Preoperative chemoradiotherapy (CRT) improves the survival of patients with oesophageal cancer when compared with surgery alone.

**Methods:**

We conducted a phase II, multicenter trial of FOLFOX-4 and cetuximab in patients with locally advanced oesophageal cancer (LAEC) followed by daily radiotherapy (180 cGy fractions to 5040 cGy) with concurrent weekly cetuximab. Cytokines levels potentially related to cetuximab efficacy were assessed using multiplex-bead assays and enzyme-linked immunosorbent assay at baseline, at week 8 and at week 17. Primary end point was complete pathological response rate (pCR).

**Results:**

In all, 41 patients were enroled. Among 30 patients who underwent surgery, a pCR was observed in 8 patients corresponding to a rate of 27%. The most frequent grade 3/4 toxicity was skin (30%) and neutropenia (30%). The 36-month survival rates were 85 and 52% in patients with pathological CR or PR *vs* 38 and 33% in patients with SD or PD.

**Conclusions:**

Incorporating cetuximab into a preoperative regimen for LAEC is feasible; no correlation between cytokines changes and patient outcome was observed. Positron emission tomography/computed tomography study even if influenced by the small number of patients appears to be able to predict patients outcome both as early and late metabolic response.

Oesophageal cancer outcome remains poor. Surgery is still the first choice of treatment for fit patients with resectable disease, but the 5-year survival is only 20–25% because lymphatic and haematogenous dissemination occurs early (Rice *et al*, 2009). Several studies suggest that preoperative chemoradiation may improve long-term outcome of resected patients when compared with surgery alone. A recent meta-analysis examined 10 randomised studies comparing trimodality therapy with surgery alone. Overall results showed a statistically significant relative reduction in mortality for patients receiving trimodality therapy with a hazard ratio of 0.81 (Lv *et al*, 2009).

Optimisation of chemoradiotherapy (CRT) using targeted therapies is a major goal of current research by increasing the rate of pathological complete response (pCR) and consequently overall survival (OS; Berger *et al*, 2005). Epidermal growth factor receptors (EGFR) expression in oesophageal cancer ranges from 30 to 70%, and it has been shown to be a poor prognostic factor (Ciardiello and Tortora, 2008). Monoclonal antibodies targeting EGFR are being extensively evaluated in several cancer types including oesophageal cancer. Data from phase III trials have demonstrated an OS benefit with the addition of cetuximab to radiation in head and neck cancer patients (Bonner *et al*, 2010).

Only one study incorporating cetuximab with CRT (carboplatin, paclitaxel, and 50.4 Gy of concurrent radiotherapy (RT)) has recently been reported in oesophageal cancer. Patients achieved an endoscopic complete response rate of 67% and, in those that underwent surgery, 43% were found to have a pCR (Sgroi *et al*, 2008). Furthermore, oxaliplatin is active and less toxic than cisplatin when administered in combination with 5-fluorouracil (5-FU) and RT in patients with locally advanced oesophageal cancer (LAEC), giving a 38% of pCR (Benjamini and Hochberg, 1995; Safran *et al*, 2008). Several studies conducted in colorectal cancer have shown the feasibility of the combination of FOLFOX-4 and cetuximab (Tabernero *et al*, 2007; Bokemeyer *et al*, 2009).

On the basis of these results, we designed a phase II multicenter trial to assess the role of cetuximab as preoperative treatment of LAEC. The potential toxicity from cetuximab concurrent with chemotherapy and RT was poorly known at the time of study design; therefore, we did not use a concurrent chemoradiation regimen with cetuximab, but we decided to use an induction chemotherapy treatment with FOLFOX-4 and cetuximab to reduce the risk of distant metastases followed by RT and cetuximab. We also evaluated, the early and late metabolic response as assessed by ^18^FDG-positron emission tomography (PET) and its correlation with objective response to treatment. Moreover, we performed explorative analyses of a pattern of cytokines and growth factors in the serum of patient before and during therapy to evaluate their potential value as predictive biomarkers for treatment outcome.

## Materials and methods

### Patient selection

Patients 18 years of age or older with locally advanced (T3–4, N0 or any T, N+) and biopsy-confirmed adenocarcinoma or squamous cell carcinoma of the oesophagus were enroled. Other eligibility criteria included Eastern Cooperative Oncology Group performance status of 0–2, no significant concomitant comorbidities; adequate organ function (absolute neutrophil count ⩾1500 cells 0 *μ*l^−1^, platelet count >100 000 *μ*l^−1^, estimated creatinine clearance >60 ml min^−1^, normal bilirubin, aspartate aminotransferase and alanine aminotransferase <1.5 × the institutional upper limit of normal (ULN), and alkaline phosphatase <2.5 × ULN. Written informed consent was obtained from all patients.

### Pre-treatment evaluation and treatment plan

Pre-treatment work-up included spiral computed tomography (CT) scans of chest and abdomen and oesophageal ultrasound endoscopic (EUS). To evaluate the correlation between metabolic response to study treatment and pathological response, on July 2008 we emended the study introducing 18 FDG-PET scan. A subset of patients was assessed by PET at the following time points: 0 (baseline), 14 days, and at week 17 (at the end of RT and before surgery). Patients were assigned to a preoperative clinical stage according to the 2002 TNM System of the American Joint Committee on Cancer. Chemotherapy consisted of oxaliplatin, 85 mg m^−2^ on day 1, folinic acid 200 mg m^−2^ as a 2 h infusion on days 1 and 2, and 5-FU, 400 mg m^−2^ bolus on days 1 and 2 followed by 5-FU 600 mg m^−2^, a 22 h continuous infusion on day 1 and 2; cycles were administered every 2 weeks. Patients received cetuximab i.v. at a starting dose of 400 mg m^−2^ followed by a weekly infusion at a maintenance dose of 250 mg m^−2^. The association of FOLFOX-4 and cetuximab was given for 8 weeks before RT. Radiation therapy was delivered using 6–20 MV X-ray of a linear accelerator. The clinical target volume contained the gross tumour with craniocaudal margins of at least 2 cm and transversal margins of 1 cm; the target volume was identified based on abnormalities observed in the oesophagus, proximal stomach and regional lymph nodes on a pre-treatment diagnostic CT scan, barium swallow and endoscopy. The dose to the spinal cord was limited to 40 Gy in all cases. A four-field conformal beam arrangement consisting of opposed anterior and posterior and lateral fields typically used. A dose of 1.8 Gy was delivered daily five times for 6 weeks up to a total dose of 50.4 Gy.

The time frame between the end of chemotherapy and the beginning of RT was 1 week.

Cetuximab was continued weekly during RT and for further 4 weeks during restaging.

Toxicity was assessed using the National Cancer Institute Common Toxicity Criteria, version 2.0. Treatment delays and dose modifications were based on the worst adverse effects observed according to previous studies (Bokemeyer *et al*, 2009; Bonner *et al*, 2010). This trial was approved by the Ethics Committee of all participating institutions and was conducted in accordance with Declaration of Helsinki, Good Clinical Practices, local and legal requirements.

### Surgery

Surgery was planned at 4 weeks after completion of restaging. Thoracic oesophageal cancers were treated with laparotomy, thoracotomy and cervicotomy followed by total oesophagectomy, lymphadenectomy and gastroesophageal anastomosis in the left neck. In abdominal oesophageal cancers, after gastric mobilisation by laparotomy, oesophagectomy was performed by right thoracotomy and mediastinal oesophagogastro anastomosis. A radical resection (R0, according to the criteria of the Union Internacional Contra la Cancrum) was defined as the removal of all macroscopic tumoural tissue, no evidence of distant metastases, the absence of microscopic residual tumour, free resection margins and lymphadenectomy extended beyond the involved nodes at post-operative pathological examination. A resection was judged as non-radical when microscopic (R1) or macroscopic (R2) residual tumour was found.

### Response assessment

Tumour response to treatment was assessed with CT scan, EUS and PET scanning after CT and RT. Systematic biopsies were required in all patients. A complete clinical response (cCR) was defined as an absence of carcinoma cells in the endoscopic biopsy and cytology specimens accompanying the disappearance of radiographic evidence of disease. A clinical partial response (cPR) was defined as a >50% regression in the volume of radiological visible tumour. Progression corresponded to either enlargement or appearance of new locoregional or distant disease. After resection, the specimens were fixed with formaldehyde and the complete tumour was embedded completely in paraffin blocks and investigated histologically. The number of paraffin blocks necessary differed with regard to the tumour size. The number of histopathological sections differed regarding the size of the specimen. The tissue was paraffin-embedded and serial sections of each block were cut (5 *μ*m) and stained with hematoxylin and eosin and periodic acid-Schiff. All specimens were classified according to the criteria of Mandard using a tumour regression grade (TRG). The TRG is based on the growth of residual tumour into the areas of adjacent fibrosis. A resection specimen with no residual tumour (complete response) is scored as TRG 1; the presence of rare residual cancer cells scattered through fibrosis is scored as TRG 2; an increased number of residual cancer cells but where fibrosis still predominates is scored as TRG 3; residual cancer outgrowing fibrosis is scored as TRG 4; and absence of regressive changes is scored as TRG 5. For the study end points, the histopathological response was divided into three groups: group 1 consisted of patients with TRG 1 (pCR), group 2 included patients with TRG 2, TRG 3 or TRG 4 (pPR), and group 3 consisted of TRG 5 (stable disease).

### Plasma collection and analyses

Plasma samples (2.5 ml) were prepared from venous blood samples collected at baseline (pre-treatment on day 1), week 8 (after chemotherapy and before RT) and week 17 (after RT and before surgery), frozen and stored at −80°C until analysis. In all, 33 molecules including growth factors, chemokines, haemopoietins were analysed by using enzyme-linked immunosorbent assay kits from R&D Systems (Minneapolis, MN, USA) and luminex analysis with multiplex beads suspension array plates (Invitrogen, Carlsbad, CA, USA). Each sample was analysed in duplicate (the complete list of assessed proteins is reported in Supplementary Material Table 1).

### Data collection and statistical analysis

Data were prospectively collected on forms to be filled out by the investigators at inclusion, after completion of the treatment sequence and at regular follow-up intervals. The primary end point of the study was pCR rate, the secondary end points were resection rate, overall survival and safety.

A two-stage Simon's mini-max design was adopted. On the basis of an *α* level of 5% and a power of 80% ‘for p0=10% and p1=25%’, 18 subjects have to be enroled at the first step of the study. In case of 2 or more with a pCR, the study would be continued until the enrolment of final sample size. Survival curves were constructed using the method of Kaplan and Meier (1958).

### Analysis of metabolic response by PET and comparison with histological response

To define the metabolic response, we applied three different cutoffs: SUV reduction of 25, 35, or 50% compared with baseline values. Therefore, patients were considered as metabolic responders when they achieved a SUV reduction of at least 25, 35 or 50%, and as non-responders when they did not achieve a reduction of at least 25, 35 or 50% of baseline SUV values (Ott *et al*, 2006).

On the basis of histological specimen results, patients were divided into histological responders (complete response/partial response) or histological non-responders (all other patients included those who did not undergo surgery because of tumour progression).

### Analysis of cytokines

Using Wilcoxon's tests, we assessed which cytokines significantly changed between different time points, specifically from baseline to intermediate and from baseline to post treatment. Given the large number of comparisons, we adjusted for multiple testing using the false discovery rate methods, which is a standard multiple test adjustment procedure (Storey, 2003). Specifically, we apply the *fdrtool* method to map each *P*-value to a *q-*value, which can be interpreted as the probability that the given factor is a false discovery (Strimmer, 2000; Storey, 2003). We identified as significant any factor with *q*<0.05.

Description of patterns of cytokines levels at baseline and during treatment according to objective response (responders *vs* non-responders) was essentially descriptive, and no formal statistical tests were performed.

## Results

### Patients characteristics

**Figure 1:**
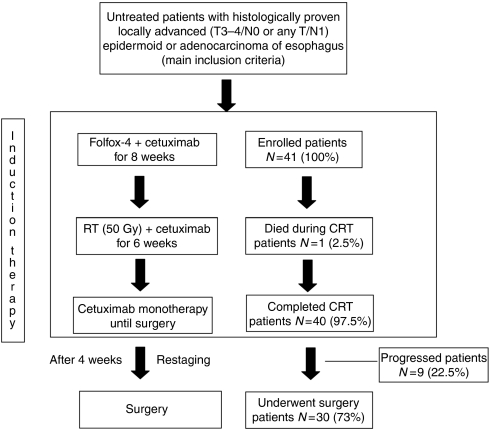
Trial design and profile.

In all, 41 eligible patients with histological verified oesophageal carcinoma were enroled between December 2006 and July 2009. Figure 1 shows the trial profile. Baseline characteristics of the study population are listed in Table 1.

### Response to chemoradiation therapy

After four cycles, dysphagia relief was observed in 94% of 35 symptomatic patients. We excluded one patient from clinical response evaluation because of early death for progression of the disease during induction treatment. Among the 40 evaluable patients, 6 had a cCR and 13 had a cPR, for an overall clinical response rate of 47.5%. A total of 12 patients were classified as stable (SD). A tumour progression (PD) was observed in nine cases: six patients experienced distant metastases only, one patient a locoregional failure only and two patients both local and distant relapse.

### Surgery

In all, 31 of the 40 patients were considered eligible for surgery, but one refused surgery although in cCR. Therefore, 30/40 patients underwent surgery and in 24/30 the resection was judged as curative with no residual disease (R0 resection rate of 80%). Six patients had microscopic residuals involving the resection margins and precluding a radical tumour resection. Two patients died after surgery with an operative mortality rate of 6%. We observed three anastomotic stenoses that needed at least one endoscopic dilatation.

A pCR (TRG1) was observed in eight patients corresponding to a rate of 20%, whereas a pPR (TRG 2, 3 and 4) was recorded in 12 patients (30%) with an overall pathological response rate of 50%. Among those patients who underwent to surgery, the pCR rate was 27%. Noteworthy, all pCR were observed in squamous cell carcinoma. Table 2 shows the treatment efficacy according to the intention to treat and in resected population.

### Survival

**Figure 2:**
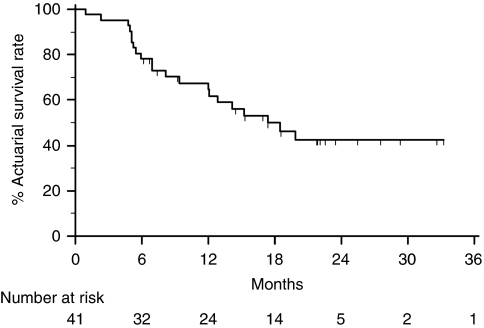
Kaplan–Meier plots of overall survival. The median and mean overall survival time was 17.3 and 16 months, respectively. The 12, 24 and 36 months overall survival rates were: 67, 42, and 42%, respectively.

All 41 patients were included in survival analysis according to the intention to treat. At the end of the study, 21 patients had died. The median and mean overall survival time was 17.3 and 16 months, respectively. The 12, 24 and 36 months overall survival rates were: 67, 42, and 42%, respectively (Figure 2). The difference in survival probability between inoperable and operable patients was significant. In fact, the 12, 24 and 36 months survival rates were 27.3, 18.2, and 18.2% in 11 non-resected patients, and 82.6, 51.1, and 51.1% in 30 resected patients, respectively (HR=3.81; 95% CI: 2.22–22.9; *P*=0.0009). The 36-month survival rates were 85 and 52% in patients with pathological CR or PR *vs* 38 and 33% in patients without pathological downstaging (SD or PD).

No differences in survival were detected among different histological type. In particular, the 3-years survival was 57% for squamous histology *vs* 41% for adenocarcinoma. *P*-value at univariate analysis was 0.5729 with HR (95% CI) 0.72 (0.21–2.34) and *P*-value at multivariate analysis of 0.3761 with HR (95% CI) of 3.65 (0.20–64.46).

### Treatment-related toxicity

Treatment-related toxicity is summarised in Table 3. In all, 40 patients completed the preoperative treatment: one patient died due to rapid progression of disease after two courses of chemotherapy. A total of 162 courses of FOLFOX-4 were administered and CT was delayed or modified in 2.9% of patients. A total of 718 courses of cetuximab were administered with a cetuximab delay or modification in 1.7% of patients. Radiotherapy was delayed or modified in 2.7% of patients. The most common grade 3 to 4 haematological and non-haematological toxicities were skin 30% and neutropenia 30%. Oesophagitis was mainly G1/G2 (77%); a G1/G2 neurotoxicity, was recorded in 47% of patients. One patient experienced a serious cervical anastomotic leak with severe mediastinitis and died at 2 months after the operation; one patient died for septic shock.

### ^18^FDG-PET

Among 41 patients enroled in this study, 11 were excluded from PET evaluation because of PET baseline assessment was not performed. Therefore, 30 resulted potentially evaluable for analysis. In all, 18 out of 30 patients underwent to 2 weeks evaluation after starting treatment and 26 patients to PET scan as planned at the end of treatment.

In 18 patients eligible for the analysis of predictive role of early metabolic response, the mean baseline SUV was 12.89 (s.d.±5.66). The mean 2 weeks SUV was 7.45 (s.d.±2.84). The mean percentage reduction from baseline was 37.8% (s.d.±19.5% *P*-value=0.0009, Wilcoxon rank sum test).

In 26 patients eligible for analysis of predictive role of post-treatment metabolic response, the baseline SUV reported a mean of 12.60% (s.d.±4.89). The mean SUV at the end of treatment was 3.80% (s.d.±3.88), with a mean percentage reduction of 63.0% (s.d.±42.8; *P*<0.001, Wilcoxon rank sum test).

### Evaluation of potential biomarkers in the plasma

In all, 33 different proteins plasma levels were measured in 28 patients at baseline, at intermediate (week 8) and post-treatment evaluation (week 17).

Table 4 describes, for each cytokine analysed, median baseline values and median changes from baseline, week 8 and at week 17. At intermediate and post-treatment evaluation, levels of several cytokines were significantly different even after multiple comparison correction, with significance defined by local false discovery rate *q*<0.05, upper limit of normal. However when these variations were evaluated according to response to treatment, there was no major difference between cytokine variation levels among responding and non-responding patients (Supplementary Material Figures 1 and 2).

## Discussion

The role of trimodality therapy of LAEC remains debated, but several data suggest a better outcome for preoperative CRT-treated patients. Despite this strategy, most of the patients develop distant metastases and die of their disease. At this time, pCR appears as an early marker of the efficacy of CRT and patients who achieve a pCR have an improved survival as shown in literature-based meta-analyses and phase II and phase III studies (Janmaat *et al*, 2006; Safran *et al*, 2008; Al-Batran *et al*, 2009). Also our previous experiences confirmed that complete pathological responder patients had a significant longer survival probability compared with patients with partial response or stable disease after preoperative CRT (De Vita *et al*, 2002; Orditura *et al*, 2010). To improve the efficacy of systemic treatments in combination with RT, a series of clinical studies are currently evaluating the addiction of molecular targeted agents with the aim of increasing the pCR; therefore, the assessment of pCR was the primary end point of this study. In this study, we report that a combined treatment consisting of 2 months of cetuximab plus FOLFOX-4 followed by 6 weekly radiation therapy plus cetuximab achieved a pCR rate of 27% in resected patients. This rate of pCR could appear lower if compared with a previous study with concurrent cetuximab, chemotherapy and RT (Safran *et al*, 2008). Nevertheless, we must underline the different study design with the choice, in the present trial, of a sequential rather than concurrent CRT approach. In fact, this trial was planned when few data were available about toxicity of concurrent RT, chemotherapy and cetuximab; this was also the reason for giving induction therapy to handle the high risk of distant metastases observed in LAEC. However, it is important to emphasise that pCRs vary in different studies from 17 to 51% with most large randomised trials demonstrating rates of pCR ranging from 10 to 30% (Apinop *et al*, 1994; Le Prise *et al*, 1994; Walsh *et al*, 1996; Bosset *et al*, 1997; Urba *et al*, 2001; Burmeister *et al*, 2005). The current pCR rate of 27% is consistent with the results of these trials. The survival observed in our study appears consistent with that observed in most large randomised trials. The median OS was 17.3 months with a 3-year OS of 42%, whereas the median OS in large randomised trials ranges from 10 to 19 months with a 3-year OS rate of 19–39% (Apinop *et al*, 1994; Le Prise *et al*, 1994; Walsh *et al*, 1996; Bosset *et al*, 1997; Urba *et al*, 2001; Burmeister *et al*, 2005). Furthermore, the present data suggest a significant survival benefit for patients experiencing a pCR, who obtained a 3-year OS rate of 85%.

One of the most important factors conditioning survival of patients undergoing oesophagectomy is an R0 resection. We obtained an R0 resection in 80% of resected patients, and this R0 resection rate compares favourably with those reported in the literature, which are typically above 80% (Hofstetter *et al*, 2002; Mariette *et al*, 2003).

This regimen was generally well tolerated. The rates and types of adverse events in our patients were consistent with those expected from the individual agents, with the exception of skin reactions due to cetuximab; 26% of patients experienced a grade 3–4 dermatological toxicity but there was not an increase in oesophagitis or other radiation-enhanced toxicity.

The PET evaluation was limited by the small number of patients. However, all the three cutoffs used (25, 35 and 50%) demonstrated, in a descriptive manner, that SUV reduction, between scans carried out at the end of treatment and the one at baseline, correlates with the response to the treatment. These data suggest that the metabolic response can be used as a parameter of patients outcome. Therefore, PET assessment of early metabolic response could be incorporated in future clinical trials of multimodality treatment for LAEC.

Finally, the explorative plasma analysis showed that protein modifications recorded at different time points respect to the basal concentrations, are not correlated with the efficacy of treatment, even for those proteins that shown a significant change (decrease or increase) in the whole population. Changes in these proteins are not strongly related to modification in tumour burden. It may be that the protein changes observed could be related to the general clinical status, to the local systemic inflammatory modifications secondary to the tumour, and/or to the body response to a combined approach such as CT and RT administered in combination with a monoclonal antibody.

In conclusion, this study shows that the combination of FOLFOX-4 and cetuximab followed by cetuximab and concurrent radiation is an active and safe preoperative regimen for LAEC.

## Figures and Tables

**Table 1:** Patient characteristics

	**No. of patients=41 (100%)**
*Age*	
Median/range	54/39–75
	
*Sex*	
Male/female	30/11 (30/27)
	
*Performance status*	
0/1	35/6 (85/15)
	
*Dysphagia*	
Absent/moderate	7/8 (17/19)
Severe	26 (63)
	
*Tumor location*	
Upper third	4 (10)
Middle third	17 (41)
Lower third	20 (49)
	
*Histology*	
Adenocarcinoma	13 (32)
Squamous cell carcinoma	28 (68)
	
*EUS T stage* a	
2	11 (27)
3	25 (62)
4	3 (7)
	
*EUS N stage* a	
0	5 (12)
1/M1a	30/4 (73/10)

Abbreviation: EUS=oesophageal ultrasound endoscopic.

a A total of 39/41 patients.

**Table 2:** Treatment activity

	**Number of patients**	**Intention to treat patients=41 (100%)**	**Patients undergoing surgery patients=30 (100%)**
Path CR	8	(19.5)	(26.6)
Path PR	12	(29.6)	(40)
Overall path RR	20	(48.7)	(66.6)
R0 surgery	24	(58.5)	(80.0)

Abbreviations: path CR=pathological response rate; path PR=pathological partial response; RR=response rate.

**Table 3:** Treatment-related toxicity

**Toxicity**	**G0 (%)**	**G1 (%)**	**G2 (%)**	**G3 (%)**	**G4 (%)**
Skin	7	13	50	30	0
Neutropenia	40	23	7	20	10
Anemia	83	12	5	0	0
Esophagitis	23	45	32	0	0
Nausea/vomiting	53	35	12	0	0
Diarrhea	85	10	5	0	0
Stomatitis	75	20	5	0	0
Neurotoxicity	53	45	2	0	0

**Table 4:** Cytokine value at baseline, at week 7 and week 17

		**Intermediate**		**Post-treatment**	
**Cytokine**	**Baseline median**	**% Baseline^a^**	***P***	**% Baseline^a^**	***P***
*Growth factors*					
VEGF	178.1	NA	NA	120	0.5891
HGF	257.5	91	0.1479	62	0.0493
FGFb	8.9	89	0.0448	80	<0.0001^b^
PlGF	38.8	108	0.927	91	0.915
Epiregulin	1.0	90	0.4807	78	0.6824
TGFa	39.8	98	0.9933	108	0.3316
EGF	29.6	53	0.3408	69	0.444
					
*Chemokines*					
Eotaxin	56.0	81	0.4527	33	0.0001^b^
IL 8	3.5	92	0.4282	72	0.1772
IP 10	8.0	148	0.0029^b^	151	0.0084^b^
MCP 1	175.5	76	0.3629	67	0.0652^b^
MIG	15.2	88	0.3598	61	0.0159^b^
MIP-1A	39.7	58	<0.0001^b^	37	<0.0001^b^
MIP-1B	47.2	58	0.0112^b^	39	<0.0001^b^
RANTES	5696.9	122	0.0724	251	0.045
					
*Haemopoietins*					
G-CSF	59.1	80	0.0014^b^	81	0.0159^b^
GM-CSF	19.3	100	0.0135^b^	103	<0.0001^b^
IL 2	1.4	99	0.2637	82	0.0201^b^
IL 2R	194.2	85	0.467	84	0.129
IL 4	27.5	49	0.0003^b^	43	<0.0001^b^
IL 5	14.1	41.3	0.2881	12	<0.0001^b^
IL 6	2.3	136	0.0164^b^	142	0.0003^b^
IL 7	11.0	142	0.0001^b^	175	<0.0001^b^
IL 13	7.2	100	0.5558	100	1
IL 15	13.3	84	0.0049^b^	63	<0.0001^b^
					
*Other molecules*					
IFNa	20.7	88	0.3498	67	<0.0001^b^
IFNg	52.6	52	<0.0001^b^	35	<0.0001^b^
TNF-A	7.3	49	<0.0001^b^	48	<0.0001^b^
IL 1b	13.3	69	0.0008^b^	57	<0.0001^b^
IL 1Ra	373.1	46	0.0002^b^	52	0.0002^b^
IL 10	1.8	183	0.001^b^	315	<0.0001^b^
IL 12	185.1	42	<0.0001^b^	21	<0.0001^b^
IL 17	13.6	82	0.1247	74	0.0044^b^

Abbreviations: EGF=epidermal growth factor; FGF=fibroblast growth factor; G-CSF=granulocyte colony-stimulating factor; GM-CSF=granulocyte–macrophage colony-stimulating factor; HGF=hepatocyte growth factor; IL=interleukin; INF=interferon; IP=inducible protein; MCP=monocyte chemoattractant protein; MIG=monokine induced by gamma interferon; MIP=macrophage inflammatory proteins; NA=not available; PlGF=placenta growth factor; RANTES=regulated on activation normal T cell expressed and secreted; TGF=transforming growth factor; TNF=tumour necrosis factor; VEGF=vascular endothelial growth factor.

a The median of the ratios of cytokine and angiogenic factor concentration at each time point to baseline concentration expressed as a percentage.

b Significant after correction for multiple comparisons (see text).
