# Supplementary figures and images for: A multicenter phase II study of induction chemotherapy with FOLFOX-4 and cetuximab followed by radiation and cetuximab in locally advanced oesophageal cancer
Source: Br J Cancer. 2011 Jan 18;104(3):427–32. doi: 10.1038/sj.bjc.6606093 (PMC3049578; doi:10.1038/sj.bjc.6606093)

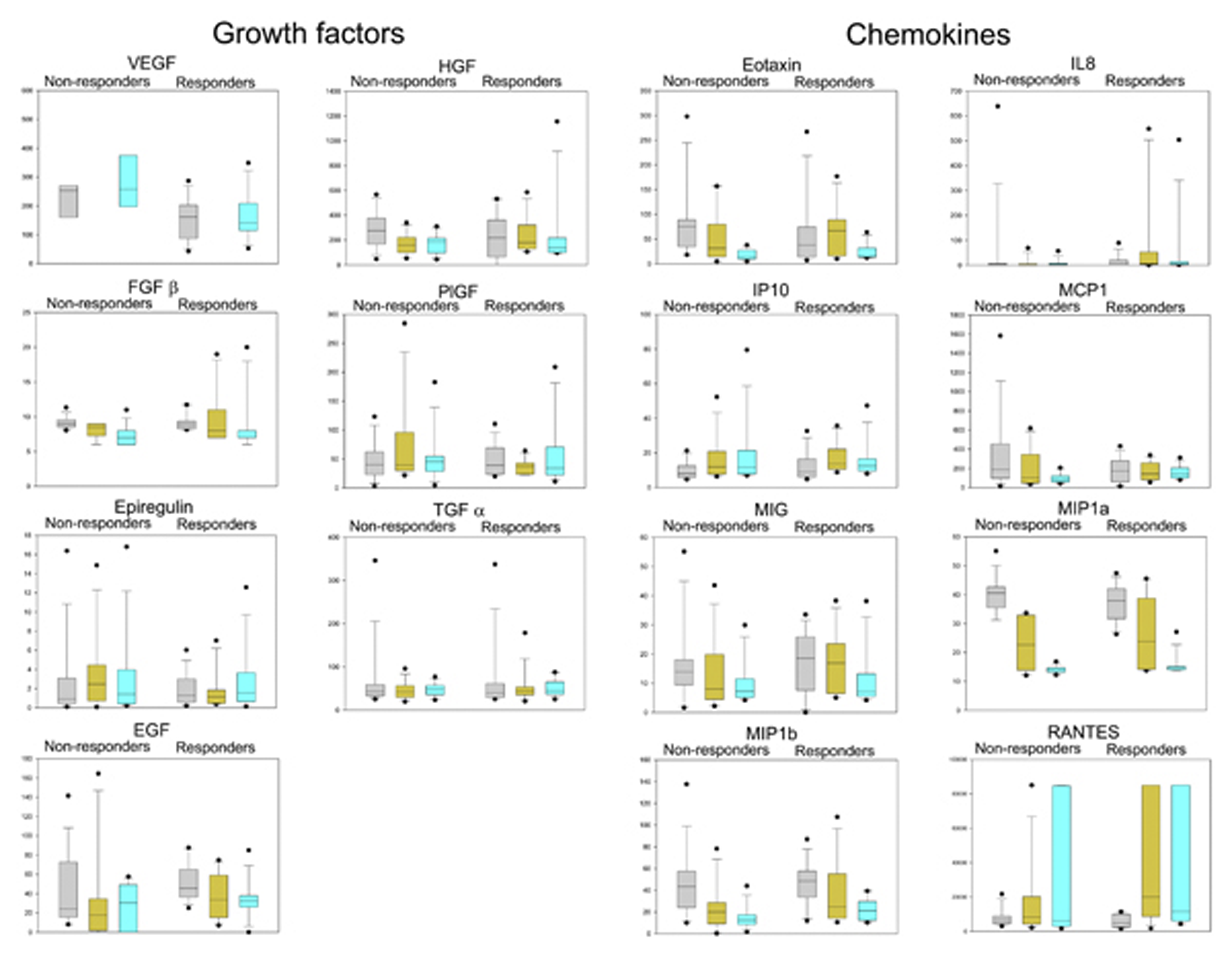

Supplement: Supplementary Figure 1 [file 6606093x1.tif]

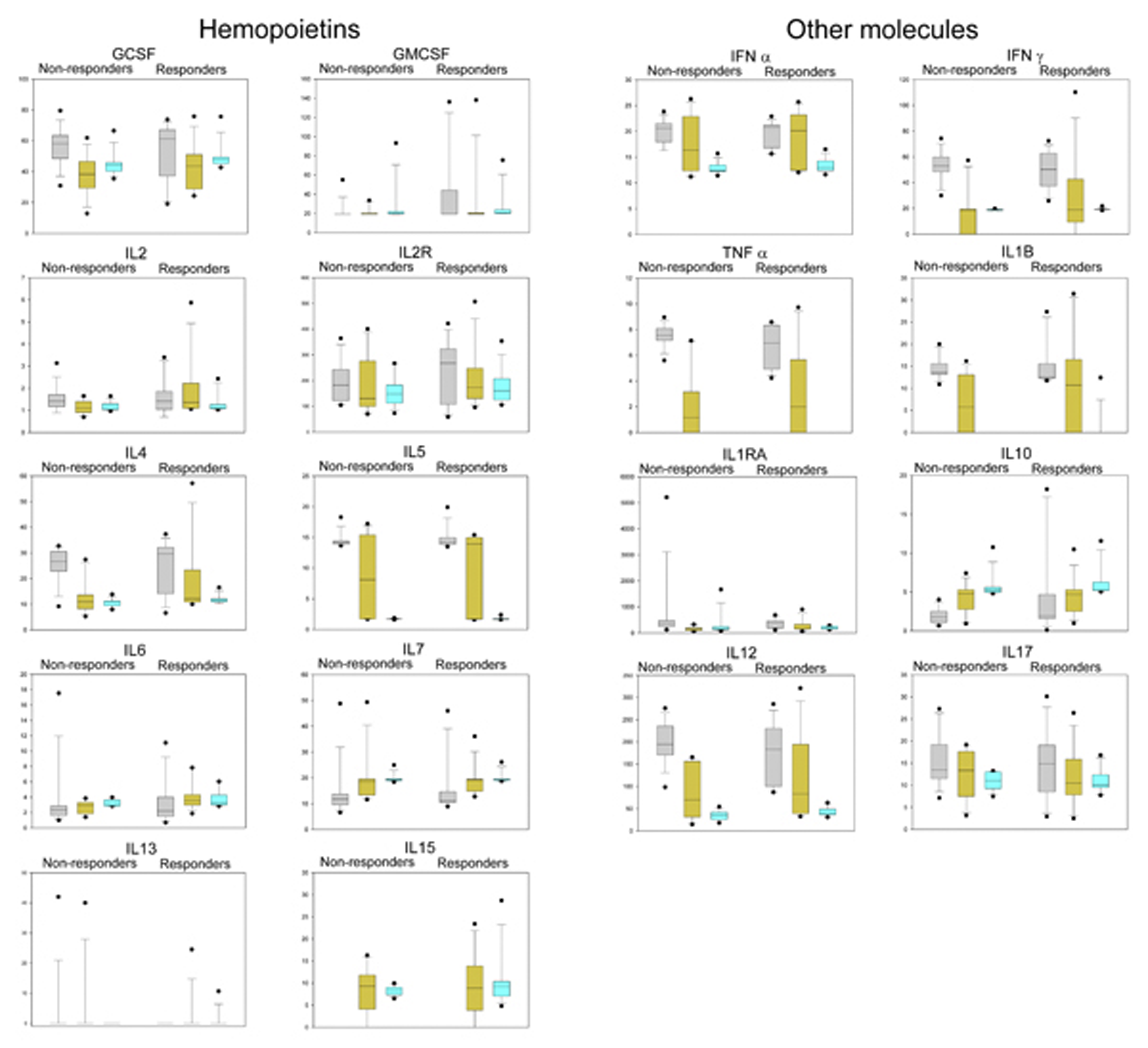

Supplement: Supplementary Figure 2 [file 6606093x2.tif]
