# Supplementary material for: A multicenter phase II study of induction chemotherapy with FOLFOX-4 and cetuximab followed by radiation and cetuximab in locally advanced oesophageal cancer
Source: Br J Cancer. 2011 Jan 18;104(3):427–32. doi: 10.1038/sj.bjc.6606093 (PMC3049578; doi:10.1038/sj.bjc.6606093)
Supplement: Supplementary Table 1 [file 6606093x3.doc]

**Supplemental Material Table 1**

**Growth factors**

VEGF

HGF

FGFb

PlGF

Epiregulin

TGFa

EGF

**Chemokines**

Eotaxin

IL8

IP 10

MCP 1

MIG

MIP-1A

MIP1-B

RANTES

**Hemopoietins**

G-CSF

GM-CSF

IL2

IL 2R

IL 4

IL 5

IL 6

IL 7

IL 13

IL 15

**Other molecules**

IFNa

IFNg

TNF-A

IL 1b

IL 1Ra

IL 10

IL 12

IL 17
