# Supplementary material for: A multicenter phase II study of induction chemotherapy with FOLFOX-4 and cetuximab followed by radiation and cetuximab in locally advanced oesophageal cancer
Source: Br J Cancer. 2011 Jan 18;104(3):427–32. doi: 10.1038/sj.bjc.6606093 (PMC3049578; doi:10.1038/sj.bjc.6606093)
Supplement: Supplementary Figure and Table Legends [file 6606093x4.doc]

**Legend to Supplemental Figures**

**Figure 1**

Variation levels of growth factors and chemokines among responding and non responding patients

**Figure 2**

Variation levels of hemopoietins and other molecules among responding and non responding patients

**Legend to Supplemental Tables**

**Table 1**

List of cytokine evaluated at baseline, at intermediate (week 8) and post-treatment evaluation at (week 17).
